# Association Between Ultraprocessed Food Intake and Self-Reported Arthritis

**DOI:** 10.1016/j.amepre.2025.02.010

**Published:** 2025-02-28

**Authors:** Yanxin Zhu, Vanessa Garcia-Larsen, Sabri Bromage, Euridice Martinez-Steele, Ana Luiza Curi-Hallal, Casey M. Rebholz, Mika Matsuzaki

**Affiliations:** 1 Department of International Health, The Johns Hopkins Bloomberg School of Public Health, Baltimore, Maryland; 2 Community Nutrition Unit, Institute of Nutrition, Mahidol University, Nakhon Pathom, Thailand; 3 Department of Nutrition, Harvard T.H. Chan School of Public Health, Boston, Massachusetts; 4 Department of Nutrition, School of Public Health, University of São Paulo, São Paulo, Brazil; 5 Departamento de Saúde Pública, Centro de Ciências da Saúde, Universidade Federal de Santa Catarina, Florianópolis, Santa Catarina, Brazil; 6 Department of Epidemiology, The Johns Hopkins Bloomberg School of Public Health, Baltimore, Maryland; 7 Welch Center for Prevention, Epidemiology and Clinical Research, Johns Hopkins University, Baltimore, Maryland

## Abstract

**Introduction:**

Ultraprocessed foods are typically high in fat, salt, sugar, and food additives, which may contribute to thedevelopment of arthritis. This study examined the association between ultra-processed food intake and the presence of self-reported arthritis.

**Methods:**

The 2001−2018 U.S. National Health and Nutrition Examination Survey data was used to analyze the association between ultraprocessed food intake and arthritis in 2025. Ultraprocessed foods were identified by applying Nova classifications to 24-hour dietary recall data and expressed as a percentage of daily total energy intake. The outcomes were self-reported physician’s diagnosis of arthritis, osteoarthritis, and rheumatoid arthritis. Survey-weighted logistic regressions were conducted to analyze associations between ultraprocessed food intake and outcomes, controlling for age, gender, race/ethnicity, smoking status, health insurance status, and poverty-to-income ratio.

**Results:**

UPFs contributed 55.2% of the daily total energy intake in the U.S. population. After adjusting for covariates, a positive association was found between daily total energy intake from ultra-processed foods and self-reported arthritis (AOR associated with each 10-percentage point increase in intake: 1.04; 95% CI=1.02, 1.06). For the second, third, and fourth quartiles of ultraprocessed food intake, the AOR of arthritis was 1.14 (95% CI=1.04, 1.25), 1.22 (95% CI=1.10, 1.35), and 1.27 (95% CI=1.14, 1.41), respectively (*p* for linear trend <0.001). A positive association was also observed between ultraprocessed food intake and rheumatoid arthritis (AOR=1.05; 95% CI=1.02, 1.09) but not for osteoarthritis.

**Conclusions:**

In this nationally representative sample of U.S. adults, higher consumption of ultra-processed foods was associated with overall arthritis and rheumatoid arthritis.

## INTRODUCTION

Arthritis is the most common cause of disability in U.S. adults.^1^ In the 2016−2018 National Health Interview Survey, an estimated 23.7% of U.S. adults reported experiencing arthritis and 10.7% of the overall U.S. population reported having arthritis-attributable ability limitations.^2^ In 2013, arthritis-related medical expenditures in the U.S. reached $303.5 billion.^3^ The 2014 estimation of the all-cause direct cost, including medical expenditure and lost wages, of osteoarthritis (OA) and rheumatoid arthritis (RA)—the two main forms of arthritis—was $373.2 and $32.9 billion, respectively.^4^ The chronic pain caused by arthritis is associated with increased anxiety, depression, and poorer quality of life.^5−7^

Dietary factors play a substantial role in the pathogenesis of arthritis,^8^ among which ultraprocessed foods (UPFs) may be of particular relevance given the ubiquity of UPF availability and consumption in the U.S. UPFs are defined as foods with the highest degree of processing according to the Nova classification system, which groups foods according to the nature, extent, and purpose of industrial processing.^9^ UPFs are often dense in caloric energy, saturated fat, and added sugar and sodium while low in dietary fiber and micronutrient concentrations.^9^ Examples of UPFs include ready-to-consume foods such as soft drinks and packaged sweet or savory snacks that often have longer shelf lives and are highly palatable by design.^10^ These characteristics have contributed to the growing consumption of UPFs in the U.S., now estimated to account for 60% of total energy intake (TEI) in the population.^11,12^ UPF consumption has been shown to be associated with a broad deterioration of diet quality in U.S. adults.^13,14^ Observational studies have found that high UPF consumption is associated with overweight and obesity, cardiometabolic diseases, depression, frailty, and all-cause mortality in adults.^15−17^ A crossover intervention trial has observed a strong association between UPF intake and increased energy intake, as well as excessive weight gain.^18^

Based on existing literature, the authors hypothesize that there are potential links between UPF intake and arthritis. High consumption of UPFs may lead to positive energy balance and interfere with immune homeostasis, contributing to metabolic and inflammatory diseases.^16^ Although different types of arthritis have varied etiologies, inflammation is a common pathway. By its nature, RA is an autoimmune inflammatory disease,^19^ while chronic inflammation is also a key process in the pathogenesis of OA.^20^ Anti-inflammatory diets have been found to prevent the development, progression, and symptoms of OA and RA.^21,22^ UPFs with high glycemic indexes are considered pro-inflammatory as they are associated with higher production of free radicals and inflammatory cytokines.^23^ Epidemiological evidence has also shown that obesity increases the risk of OA in both load-bearing and non−load-bearing joints.^24^ However, the impact of obesity on arthritis development goes beyond its weight-related effects, as obesity is associated with other risk factors for arthritis, such as abnormal blood glucose and lipid metabolisms.^25−27^ Obesity may also exacerbate arthritis through mechanical and immune-mediated mechanisms.^28^ *In vitro* and *in vivo* studies suggested that adipose tissue mediates the progression of OA through the release of adipokines and other cytokines,^29,30^ which may produce systemic inflammation in obesity and increase the likelihood of arthritis. Finally, UPFs may also alter gut microbiome diversity, particularly the abundance of *Prevotella* species, subsequently inducing inflammation and leading to the development of arthritis.^31−33^

Few existing studies have investigated the association between UPF intake and arthritis. This study analyzed population-based data from the U.S. National Health and Nutrition Examination Survey (NHANES) to examine the association between UPF intake and any arthritis among adults as well as specific types of arthritis.

## METHODS

### Population

NHANES is an ongoing, repeated cross-sectional survey of noninstitutionalized civilians conducted by the U.S. Centers for Disease Control and Prevention.^34^ NHANES employs a multistage probability design to collect data on diverse health and nutrition indicators from nationally representative samples of the U.S. population. The survey has been conducted on an annual basis since 1999. All participants provided written informed consent, and all NHANES protocols were approved by the National Center for Health Statistics ethics review board.

**Figure 1.**
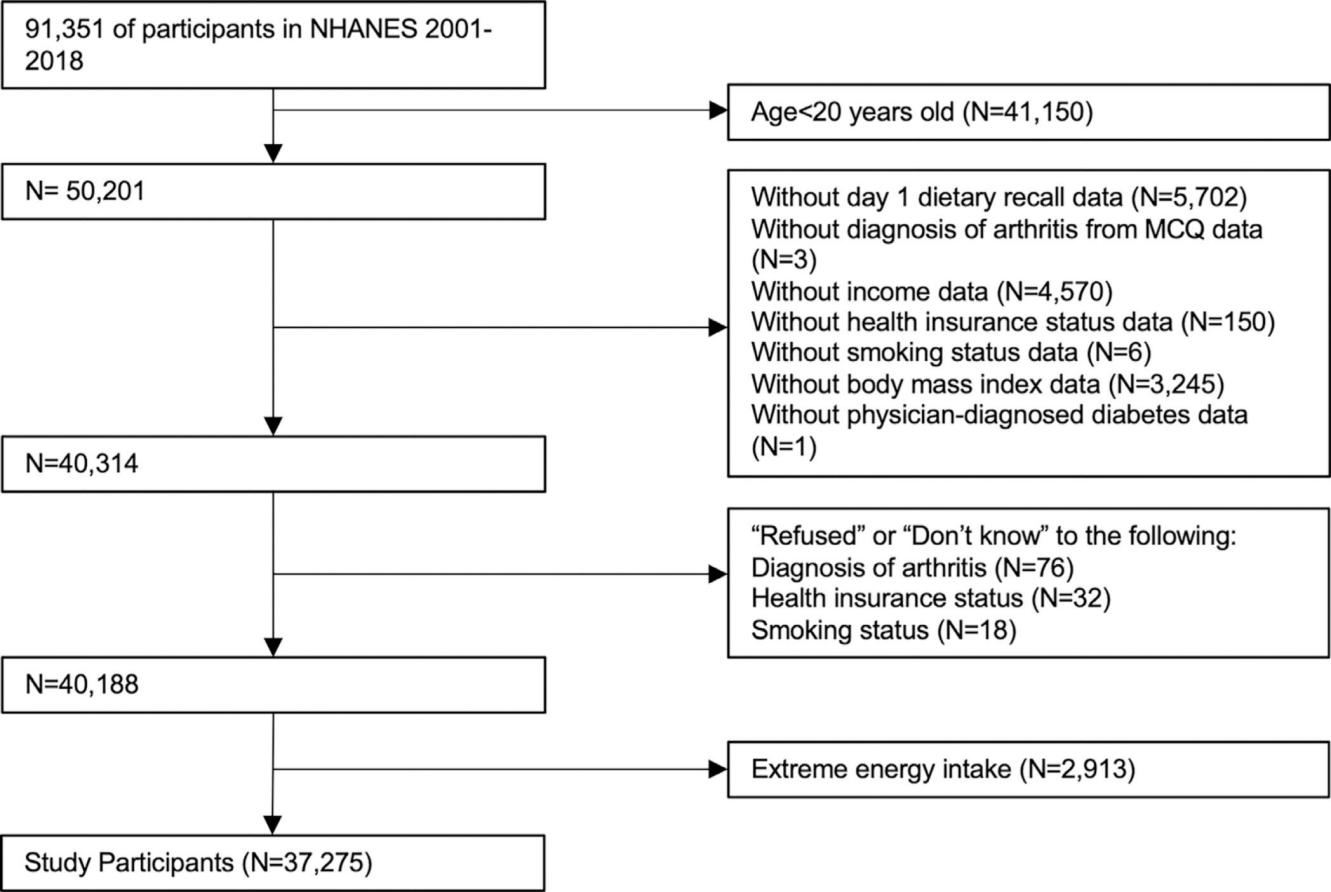
Flowchart of the selection of the analytical sample from the 2001−2018 National Health and Nutrition Examination Survey data. MCQ, Medical Condition Questionnaire; NHANES, National Health and Nutrition Examination Survey.

This study analyzed data from NHANES 2001−2018. All data were obtained from secondary sources and available publicly. No protocol approval was necessary. Participants were excluded if they were aged under 20 years (arthritis data were collected from those who were aged 20 years and above) or had missing data on physician-diagnosed arthritis or diabetes, the first-day dietary recall, family poverty-to-income ratio (PIR), health insurance status, BMI, or smoking history. The authors also excluded participants with extreme energy intake (outside the range of 800−4000 kcal/day for males or 500−3500 kcal/day for females)^35^ and those who answered *Refused* or *Don*’*t know* to questions on arthritis diagnosis, health insurance, and smoking. The participant flow chart is shown in Figure 1. Survey weights were drawn from the first-day dietary recall file and recalculated in pooling across survey waves.^36^

### Measures

Types and amounts of foods and beverages consumed in the last 24 hours were measured by trained interviewers using the U.S. Department of Agriculture Automated Multi-Pass Method on 2 recall days.^37^ The first recall was in-person at a Mobile-Examination Center and the second over the phone 3−10 days later. For this study, data from the first day of recall were used, as the second-day diet was not assessed in the 2001−2002 cycle and a relatively large proportion of participants (12.5%) had missing data for the second day in the subsequent cycles. The Nova system was applied to classify UPFs according to published guidance.^38^ Energy intake from each food consumed, available from NHANES and computed using the Food and Nutrient Database for Dietary Studies,^39^ was used to compute daily energy intake from UPFs and the percentage of total energy intake (%TEI) from UPFs. In this analysis, %TEI from UPFs was analyzed as both a continuous variable (for every 10% increase) and as quartiles of the pooled study population (2001−2018) to evaluate potential nonlinear trends in associations. To capture the intake of zero- or low-calorie UPFs, UPF consumption measured as a percentage of total diet grams/day (% grams/day) was also used.

In the Medical Condition Questionnaire, study participants were asked, *Has a doctor or other health professional ever told you that you had arthritis?* In the case of an affirmative response, participants were asked, *Which type of arthritis was it?* Possible answers included: (1) RA, (2) OA or degenerative arthritis, (3) other types of arthritis, or (4) refused or don’t know. The outcomes of this study were overall arthritis (doctor-diagnosed arthritis), RA, and OA.

Covariates included age, self-reported gender (men/women), race/ethnicity (Mexican American, other Hispanic, non-Hispanic White, non-Hispanic Black, and other races), PIR, health insurance status, and smoking status. PIR was the ratio of family income to the U.S. poverty threshold defined for each year; it was grouped into 2 categories (sub-poverty income versus other) using a cut-point of 130%.^40^ Health insurance status was defined as answering *yes* to *Are you covered by health insurance or some other kind of health care plan?* Smoking status was stratified into three groups: people who never smoked (<100 cigarettes in life), people who used to smoke, and people who smoke currently. BMI was considered a potential mediator in the UPF-arthritis relationship and was not adjusted for in main regression models. Weight status was categorized as underweight or normal (<25 kg/m^2^), overweight (25 kg/m^2^≤BMI<30 kg/m^2^), or obese (≥30 kg/m^2^).^41^ Central obesity using waist circumference (≥102 cm for males and ≥88 cm for females) was also employed.^42^ Diabetes was defined as those who answered *yes* or *borderline* for medical diagnosis of diabetes. Given the potential bidirectional relationships between diabetes and arthritis, diabetes was included in the sensitivity analyses only.^43^

### Statistical Analysis

Survey-weighted descriptive statistics were calculated to describe the characteristics of the study population and the distribution of arthritis prevalence and UPF intake by selected covariates. Chi-square tests were used to compare the distribution of arthritis across population subgroups, and the Cochran−Armitage trend test evaluated trends in prevalence across quartiles of UPF consumption within subgroups. Univariable and multivariable-adjusted survey-weighted logistic regression models were used to investigate associations between %TEI from UPFs and study outcomes in the total population and stratified by BMI categories, controlling for age, gender, race/ethnicity, PIR, health insurance status, and smoking status. Tests for linear trends in associations across quartiles of UPF intake were carried out, including quartiles as a continuous ordinal variable. Interactions of continuous BMI, gender, and diabetes status were assessed. Sensitivity analyses were performed, including UPFs in %grams/day, further adjusting for continuous BMI, central obesity, diabetes, and survey waves. Stratified analyses by gender and diabetes status were also done. Statistical analyses were performed using R version 4.4.2 (R Core Team, Vienna, Austria) in 2025.

## RESULTS

The final analytical sample size was 37,275 before weighting. The mean age of the study population was 47.4±0.2 years. The prevalence of arthritis was overall 25.8%, including RA 4.0%, OA 11.3%, other arthritis 3.2%, and 7.3% who refused or didn’t know their arthritis types (Table 1). Overall arthritis, RA, and OA were more prevalent among women, those covered by health insurance, people who used to smoke and who smoke currently, and those with central obesity (*p*<0.001). More RA cases were reported in non-Hispanic Black individuals and those without diabetes, while a higher prevalence of overall arthritis and OA was observed in non-Hispanic White individuals and those with diabetes (all *p*<0.001). The prevalence of overall arthritis and OA increased over time (*p*=0.008). The prevalence of all three outcomes increased with older age (*p*<0.001) and higher BMI (*p*<0.001). There was no clear association between overall arthritis and PIR (*p*=0.16), but more RA cases were observed in low PIR and the opposite for OA (*p*<0.001). Appendix Table 1 presents unweighted descriptive statistics for the study participants.

The median percentage of energy intake from UPFs in the U.S. population was 55.2% (IQR: 40.8%−69.4%). From 2001−2006 to 2013−2018, the percentage of individuals in the highest and lowest quartiles of UPF intake increased (Table 2). The youngest participants, women, non-Hispanic White individuals, non-Hispanic Black individuals, lower PIR, no health insurance, people who smoke currently, without diabetes, BMI≥30 kg/m^2^, without central obesity, with arthritis, and RA tended to be more concentrated in the higher UPF quartiles.

In the fully adjusted model, each 10-percentage point higher %TEI from UPFs was associated with a 4% higher likelihood of having any type of arthritis (AOR=1.04; 95% CI=1.02, 1.06) (Table 3). For RA, each 10-percentage point higher intake of %TEI from UPFs was associated with a 5% higher likelihood of RA in multivariable analysis (AOR=1.05; 95% CI=1.02−1.09). The AORs for having RA across quartiles of %TEI from UPF intake were 1.20 (95% CI=0.97, 1.49), 1.29 (95% CI=1.06, 1.57), and 1.38 (95% CI=1.13, 1.67) (*p*-trend=0.001). When analyses of RA were stratified by BMI category, each 10-percentage point higher intake of %TEI from UPFs was associated with 7% and 5% higher possibility of being diagnosed with RA among individuals with 25≤BMI<30 and BMI≥30 respectively. The AORs for having RA for those with 25≤BMI<30 across Q2-Q4 were 1.50 (95% CI=1.07, 2.10), 1.33 (95% CI=0.96, 1.84), 1.57 (95% CI=1.16, 2.13) (*p*-trend=0.01), and for BMI≥30 1.08 (95% CI=0.76, 1.53), 1.35 (95% CI=1.00, 1.83), 1.30 (95% CI=0.97, 1.74) (*p*-trend=0.03). No clear association was observed in the analysis of OA or other types of arthritis.

Appendix Table 2 presents the results from the sensitivity analyses. When UPF intake was measured in %grams/day, similar positive associations were observed for overall arthritis (AOR=1.38; 95% CI=1.17, 1.63) and RA (AOR=1.63; 95% CI=1.18, 2.24) but not for OA. The remaining analyses used UPF (%TEI) to assess the UPF-arthritis relationship with further adjustments or stratifications. There was no clear evidence of effect modification by gender (*p*=0.63, 0.44, and 0.77), continuous BMI (*p*=0.09, 0.70, 0.55), or diabetes (*p*=0.36, 0.99, and 0.34) on the effects of the association between UPF and any outcome. Further adjustment of continuous BMI, central obesity, and diabetes status slightly lowered the AOR for overall arthritis and RA (all *p*<0.05). Additional adjusting for survey waves did not change the effects of UPF consumption. Positive associations were found in both genders for overall arthritis but only in women for RA (all *p*<0.05). A positive association was found in participants without diabetes between UPF intake and RA (AOR=1.06; 95% CI=1.02, 1.10) and in overall arthritis regardless of diabetes status (*p*<0.05).

## DISCUSSION

In the U.S. adult population, a positive dose-response association between UPF intake and overall arthritis was observed. This association was seen for RA specifically but not for OA. To the authors’ knowledge, this study is the first to examine the association between UPF intake and arthritis in a nationally representative sample of the U.S. population.

Previous studies have linked UPF consumption with the known risk factors for arthritis. Numerous studies have found associations between high levels of UPF consumption and excess weight and adiposity gain, with one small crossover trial showing UPF-rich diets led to a 508 kcal increase in energy intake and 0.9 kg weight gain over just 2 weeks of controlled feeding.^18,44,45^ A meta-analysis of eleven U.S. and European studies showed a strong dose-response relationship between BMI and RA.^46^ This is consistent with the findings in this study where there was a positive association between UPF and RA.

Direct or indirect impacts of UPFs through obesity and microbiome changes on inflammation may contribute to the development of arthritis. Another NHANES study provided evidence that systematic inflammation was associated with RA.^47^ A retrospective UK Biobank study found that greater UPF intake was associated with a 17% increased risk for RA, potentially mediated by inflammation.^48^ Recent studies have pointed to the potentially detrimental effects of UPFs on microbiota composition through mechanisms such as dysbiosis and reduced diversity.^49−51^ Gut microbiota, on the other hand, influences the development of arthritis through various pathways. Expansion of *Prevotella* species was found in patients with early RA,^52,53^ and animal and human studies support the pro-inflammatory properties of *Prevotella copri*, a likely cause of RA onset.^54,55^ Metabolic endotoxemia from lipopolysaccharides and microbiota-derived metabolites such as trimethylamine-N-oxide trigger inflammation and could ultimately result in hand OA.^56^

Food additives, salt, and sugar are prevalent in UPFs and may contribute to a higher risk for RA.^8,57^ Colorants and anticaking components have oxidative stress-inducing abilities, which may lead to gut microbiota alterations and eating behavior disorders.^57^ Emulsifiers may influence gut microbiome function through bacteria adherence and translocation, increasing the risk for inflammatory diseases, such as inflammatory bowel disease.^58^ High levels of salt and simple sugar in UPFs also contribute to increased C-reactive protein levels,^23^ and epidemiological studies have found that excessive consumption of sugar-sweetened beverages was crucial for RA development.^59^ High salt diet has also been shown to activate inflammatory pathways by reducing *Lactobacillus* in gut microbiota.^60^

While there are different types of UPFs, they might pose varying effects on arthritis onset. Evidence suggests that food groups like sugar-sweetened beverages, alcohol, and pizza are associated with higher risks for arthritis,^61−63^ but dairy products might be protective.^64^ Previous studies have investigated whether specific processed food groups and arthritis outcomes with mixed findings. A cohort study in Demark found no association between red processed meat and fiber intake and chronic inflammatory diseases (including RA and psoriatic arthritis).^65^ A Swedish cohort study with a follow-up time of 12 years also detected no association between the consumption of meat or meat products (including processed meat) and RA in females.^66^ A prospective study using the UK Biobank data discovered an inverse association between breakfast cereals and RA, but this association nullifies if the cereal was highly processed.^67^

This study did not reveal a relationship between UPF consumption and OA in the overall U.S. population or any BMI category. To the extent that the mechanisms by which UPF intake drives the development of arthritis are inflammatory rather than degenerative in nature, it is also possible that RA is more susceptible to UPF intake than OA. However, a few studies found a positive association between UPF intake and knee OA in the U.K.^68^ and among females in the U.S.^69^ A positive association between UPFs and overall arthritis and RA was also revealed among participants without diabetes but not those with diabetes, suggesting that diet affects arthritis more directly among those without diabetes possibly by increasing inflammation significantly.

### Limitations

This study used a nationally representative survey over 19 years of analysis, employing a validated, computer-assisted dietary assessment platform and physician diagnosis of arthritis. However, the ability to detect true associations between diet and arthritis could be affected by errors in self-reporting physician diagnoses as well as the short reference period (24 hours) over which diet was assessed (by comparison, arthritis develops over years or decades), and that one 24-hour dietary recall may not capture usual intakes. Excluding missing covariates might bias the association downward, especially for lower PIR and higher BMI categories, which would have been associated with higher odds of arthritis. Nevertheless, with low proportions of missingness (1.6% and 7.6% for BMI and PIR, respectively), the deviation of this study’s estimate from the true OR is likely to be small. Furthermore, as a cross-sectional study, this study cannot identify a temporal relationship between UPF intake and the risk of arthritis. UPF miss-classification—though likely nondifferential unless people with arthritis tend to underreport UPF intakes due to social desirability bias—could dilute the associations towards the null. Although some literature presents that the pain level and functionality changes in OA are lower than in RA,^70,71^ some evidence suggests that OA symptoms are as severe as or worse than RA.^72,73^ It is therefore plausible that some participants diagnosed with OA are more likely to shift toward healthier diets to reduce the intake of UPFs, which may partly explain the null findings for OA. Prospective cohort studies, employing adequately long periods of observation, are warranted to better elucidate the possible causal relationship between UPFs and different types of arthritis, along with formal mediation analysis for BMI.

## CONCLUSIONS

In the U.S. adult population, higher consumption of ultraprocessed foods was associated with prevalent arthritis, RA in particular. Given the high prevalence and burden of arthritis and its associated burden in the U.S. population, prospective studies on the role of UPFs in the development of arthritis and its associated symptoms are warranted.

## Supplementary Material

MMC1

SUPPLEMENTAL MATERIAL

Supplemental materials associated with this article can be found in the online version at https://doi.org/10.1016/j.amepre.2025.02.010.

## Figures and Tables

**Table 1.** Characteristics of U.S. Adults in National Health and Nutrition Examination Survey 2001–2018 Data (N=187,820,589)^a^

Variables	Total, n (%)	Arthritis (%)	*p-*value^c^	RA (%)	*p-*value^c^	OA (%)	*p-*value^c^

Survey waves^b^			**0.008**		0.51		**<0.001**
2001–2006	175,818,007 (31.2)	24.3		4.3		8.8	
2007–2012	188,531,924 (33.5)	25.0		4.0		10.1	
2013–2018	199,111,836 (35.3)	27.9		3.9		14.6	
Age (years)	47.4 (0.2)^d^		**<0.001**		**<0.001**		**<0.001**
20−44	86,136,568 (45.9)	8.7		1.6		2.7	
45−64	67,284,614 (35.8)	33.0		5.5		13.9	
≥65	34,399,407 (18.3)	54.6		7.3		27.5	
Gender			**<0.001**		**<0.001**		**<0.001**
Men	87,175,883 (46.4)	21.5		3.5		8.3	
Women	100,644,706 (53.6)	29.6		4.5		13.8	
Race/ethnicity			**<0.001**		**<0.001**		**<0.001**
Mexican American	14,891,417 (7.9)	13.6		3.3		3.9	
Other Hispanic	9,184,704 (4.9)	17.4		4.1		5.2	
Non-Hispanic White	130,528,922 (69.5)	28.8		3.9		13.6	
Non-Hispanic Black	20,206,038 (10.8)	23.2		6.4		6.3	
Other races	13,009,508 (6.9)	19.2		3.0		8.5	
PIR			0.16		**<0.001**		**<0.001**
0%–129%	39,590,623 (21.1)	26.8		6.1		9.0	
≥130%	148,229,966 (78.9)	25.5		3.5		11.9	
Health insurance status			**<0.001**		**<0.001**		**<0.001**
No	31,266,881 (16.6)	13.3		2.8		4.1	
Yes	156,553,708 (83.4)	28.3		4.3		12.7	
Smoking status			**<0.001**		**<0.001**		**<0.001**
People who never smoke	101,704,296 (54.1)	21.6		3.0		9.9	
People who used to smoke	47,165,241 (25.1)	35.1		5.1		16.6	
People who smoke currently	38,951,052 (20.7)	25.6		5.4		8.6	
Diabetes			**<0.001**		**<0.001**		**<0.001**
No	167,111,230.50 (89.0)	23.2		27.0		10.2	
Yes	20,660,284.06 (11.0)	47.2		7.9		19.8	
Don’t know	49,074.46 (0.0)	16.3		6.4		3.8	
BMI (kg/m^2^)			**<0.001**		**<0.001**		**<0.001**
<25	57,392,882 (30.6)	18.3		3.1		7.8	
25≤BMI<30	62,261,781 (33.1)	24.4		3.5		11.0	
≥30	68,165,925 (36.3)	33.4		5.3		14.5	
Central obesity			**<0.001**		**<0.001**		**<0.001**
Yes	103,256,190 (56.3)	32.6		5.0		14.5	
No	80,274,835 (43.7)	16.3		2.8		6.7	
Type of arthritis			*NA*		*NA*		*NA*
RA	7,592,788 (4.0)	*NA*		*NA*		*NA*	
OA	21,181,391 (11.3)	*NA*		*NA*		*NA*	
Others	5,919,945 (3.2)	*NA*		*NA*		*NA*	
Refused or don’t know	13,776,392 (7.3)	*NA*		*NA*		*NA*	

Note: Boldface indicates statistical significance (*p*<0.05).

PIR, poverty-to-income ratio; RA, rheumatoid arthritis; OA, osteoarthritis.

a All statistics survey-weighted. Values in columns No and Yes expressed as %.

b Indicates that survey weights were calculated separately for each survey wave.

c *p*-values estimated using Chi-square tests.

d This is mean ± standard error.

**Table 2.** Distribution of Characteristics of the U.S. Population Across Quartiles of Ultraprocessed Food Intake (N=187,820,589)^a,b^

Variables	Q1 (%) n=46,954,497	Q2 (%) n=46,942,895	Q3 (%) n=46,976,547	Q4 (%) n=46,946,650

Survey waves				
2001–2006	24.8	26.5	27.2	21.6
2007–2012	24.0	24.7	24.9	26.4
2013–2018	26.1	24.0	23.3	26.7
Age (years)				
20–44	23.1	23.5	24.8	28.5
45–64	26.2	25.4	25.2	23.3
≥65	27.4	27.9	25.2	19.5
Gender				
Men	25.6	25.9	24.5	24.0
Women	24.5	24.2	25.4	25.8
Race/ethnicity				
Mexican American	26.0	28.1	26.4	19.4
Other Hispanic	36.1	26.8	19.9	17.2
Non-Hispanic White	22.6	25.3	26.0	26.1
Non-Hispanic Black	21.6	22.3	25.3	30.8
Other races	45.6	21.4	16.7	16.4
PIR				
0%–129%	25.9	22.2	23.3	28.6
≥130%	24.8	25.7	25.5	24.0
Health insurance status				
No	25.5	23.4	24.2	27.0
Yes	24.9	25.3	25.2	24.6
Smoking status				
People who never smoke	25.5	25.4	25.2	23.9
People who used to smoke	26.0	26.5	24.9	22.6
People who smoke currently	22.5	22.0	24.8	30.7
Diabetes				
No	25.1	24.8	25.0	25.0
Yes	23.7	26.3	25.0	24.9
Don’t know	60.3	0.0	10.6	29.1
BMI (kg/m^2^)				
<25	28.2	25.2	23.6	23.0
25≤BMI<30	26.2	26.4	24.2	23.1
≥30	21.2	23.5	26.9	28.4
Central obesity				
Yes	27.9	25.3	23.9	22.9
No	22.8	24.7	25.9	26.5
Arthritis				
No	25.4	24.8	24.7	25.1
Yes	23.9	25.6	26.0	24.6
Type of Arthritis^c^				
RA	21.6	25.0	26.3	27.2
OA	25.2	25.4	25.6	23.8
Other	22.1	25.4	28.2	24.3
Refused or son’t know	23.8	26.3	25.3	24.6

OA, osteoarthritis; PIR, poverty-to-income ratio; RA, rheumatoid arthritis.

a All statistics survey-weighted and expressed as %. Q1-Q4 indicate quartiles of ultraprocessed food (UPF) intake expressed as a percentage of total energy intake (%TEI): Q1: %TEI≤40.79, Q2: 40.79<%TEI≤55.17, Q3: 55.17<%TEI≤69.41, Q4: %TEI>69.41.

b Cochran−Armitage trend tests were performed across quartiles within population characteristics (*p*<0.001 for all trends).

c These statistics are calculated among individuals with arthritis only (n=48,470,515).

**Table 3.** Associations Between Percentage of Total Energy Intake (%TEI) From Ultraprocessed Foods (UPFs) and Arthritis^a,b^

Models	Q1	Q2	Q3	Q4	*p-*trend	Per 10% TEI, *p*-value

Overall arthritis						
Model 1	Ref	1.10 (1.01, 1.19)	1.12 (1.02, 1.22)	1.04 (0.95, 1.15)	0.38	1.00 (0.99, 1.02), 0.55
Model 2	Ref	1.12 (1.02, 1.23)	1.22 (1.10, 1.35)	1.32 (1.18, 1.47)	**<0.001**	1.05 (1.03, 1.07), **<0.001**
Model 3	Ref	1.10 (1.01, 1.19)	1.11 (1.02, 1.21)	1.03 (0.94, 1.13)	0.54	1.00 (0.99, 1.02), 0.76
Model 4	Ref	1.13 (1.03, 1.24)	1.22 (1.10, 1.35)	1.31 (1.18, 1.46)	**<0.001**	1.05 (1.03, 1.07), **<0.001**
Model 5	Ref	1.14 (1.04, 1.25)	1.22 (1.10, 1.35)	1.27 (1.14, 1.41)	**<0.001**	1.04 (1.02, 1.06), **<0.001**
*Model 5 stratified by BMI (kg/m^2^)* ^d^						
<25	Ref	1.05 (0.86, 1.29)	1.10 (0.88, 1.36)	1.14 (0.93, 1.41)	0.18	1.02 (0.98, 1.05), 0.30
25≤BMI<30	Ref	1.11 (0.95, 1.31)	0.97 (0.82, 1.16)	1.22 (1.02, 1.45)	0.12	1.03 (0.99, 1.06), 0.21
≥30	Ref	1.12 (0.94, 1.33)	1.28 (1.08, 1.52)	1.13 (0.94, 1.35)	0.13	1.02 (0.99, 1.06), 0.19
RA						
Model 1	Ref	1.17 (0.95, 1.43)	1.23 (1.01, 1.49)	1.27 (1.05, 1.55)	**0.02**	1.04 (1.01, 1.07), **0.02**
Model 2	Ref	1.17 (0.95, 1.44)	1.27 (1.05, 1.55)	1.46 (1.19, 1.76)	**<0.001**	1.07 (1.03, 1.10), **<0.001**
Model 3	Ref	1.17 (0.95, 1.44)	1.22 (1.01, 1.48)	1.26 (1.04, 1.54)	**0.02**	1.04 (1.00, 1.07), **0.03**
Model 4	Ref	1.17 (0.95, 1.44)	1.27 (1.05, 1.54)	1.44 (1.18, 1.75)	**<0.001**	1.07 (1.03, 1.10), **<0.001**
Model 5	Ref	1.20 (0.97, 1.49)	1.29 (1.06, 1.57)	1.38 (1.13, 1.67)	**0.001**	1.05 (1.02, 1.09), **0.002**
*Model 5 stratified by BMI (kg/m^2^)*						
BMI<25	Ref	1.03 (0.69, 1.53)	0.93 (0.60, 1.44)	1.04 (0.65, 1.67)	0.98	0.99 (0.92, 1.08), 0.97
25≤BMI<30	Ref	1.50 (1.07, 2.10)	1.33 (0.96, 1.84)	1.57 (1.16, 2.13)	**0.01**	1.07 (1.01, 1.13), **0.02**
BMI≥30	Ref	1.08 (0.76, 1.53)	1.35 (1.00, 1.83)	1.30 (0.97, 1.74)	**0.03**	1.05 (1.00, 1.10), **0.05**
OA						
Model 1	Ref	1.01 (0.89, 1.14)	1.02 (0.89, 1.16)	0.94 (0.81, 1.09)	0.46	0.99 (0.97, 1.01), 0.43
Model 2	Ref	1.01 (0.88, 1.15)	1.08 (0.94, 1.24)	1.15 (0.98, 1.35)	0.07	1.03 (1.00, 1.06), **0.04**
Model 3	Ref	1.01 (0.89, 1.14)	1.01 (0.88, 1.15)	0.92 (0.80, 1.07)	0.33	0.99 (0.96, 1.01), 0.29
Model 4	Ref	1.02 (0.89, 1.17)	1.08 (0.93, 1.24)	1.14 (0.97, 1.34)	0.10	1.03 (1.00, 1.06), **0.05**
Model 5	Ref	1.01 (0.88, 1.16)	1.06 (0.92, 1.23)	1.12 (0.95, 1.31)	0.16	1.02 (1.00, 1.05), 0.11
*Model 5 stratified by BMI (kg/m^2^)*						
BMI<25	Ref	1.09 (0.83, 1.44)	1.01 (0.75, 1.38)	0.87 (0.63, 1.20)	0.34	0.97 (0.92, 1.03), 0.33
25≤BMI<30	Ref	0.92 (0.73, 1.16)	0.88 (0.71, 1.10)	1.20 (0.91, 1.58)	0.25	1.03 (0.99, 1.09), 0.15
BMI≥30	Ref	0.97 (0.76, 1.23)	1.09 (0.87, 1.37)	1.01 (0.79, 1.28)	0.71	1.01 (0.97, 1.05), 0.80

Note: Boldface indicates statistical significance (*p*<0.05).

Model 1: Outcome ~ UPF (per 10% TEI).

Model 2: Outcome ~ UPF (per 10% TEI) + age.

Model 3:Outcome ~ UPF (per 10% TEI) + gender.

Model 4:Outcome ~ UPF (per 10% TEI) + age + gender.

Model 5:Outcome ~ UPF (per 10% TEI) + age + gender + race/ethnicity + poverty-to-income ratio + health insurance status + smoking status. RA, rheumatoid arthritis; TEI, total energy intake; OA, osteoarthritis.

a Cells indicate survey-weighted ORs (95% CIs) and *p-*values.

b Q2-Q4 indicate quartiles of ultraprocessed food (UPF) intake expressed as a percentage of total energy intake (%TEI) (Q1: reference group). Q1: %TEI≤40.79, Q2: 40.79<%TEI≤55.17, Q3: 55.17<%TEI≤69.41, Q4: %TEI>69.41.

d *p*=0.09,0.70, 0.55 for the interaction between UPF intake and continuous BMI for arthritis, RA and OA, respectively.
