# Supplementary material for: Association Between Ultraprocessed Food Intake and Self-Reported Arthritis
Source: Am J Prev Med. Author manuscript; Available in PMC 2025 Nov 20. (PMC12632185; doi:10.1016/j.amepre.2025.02.010)
Supplement: MMC1 [file NIHMS2062286-supplement-MMC1.pdf]

Association Between Ultra-Processed Food Intake and Self-Reported Arthritis

Yanxin Zhu, Vanessa Garcia-Larsen, Sabri Bromage, Euridice Martinez-Steele, Ana Luiza Curi-Hallal, Casey M. Rebholz, Mika Matsuzaki

American Journal of Preventive Medicine

Appendix Table 1. Characteristics of US adults from National Health and Nutrition Examination Survey 2001-2018 data (N=37,275).[a]

Variables	Total, n (%)	Arthritis (%)	*P* value[b,c]	RA (%)	*P* value[b,c]	OA (%)	*P* value[b,c]
**Survey waves**			**<0.001**		0.62		**<0.001**
2001-2006	11,605 (31.1)	25.8		5.1		8.5	
2007-2012	13,390 (35.9)	27.7		5.4		9.6	
2013-2018	12,280 (32.9)	27.9		5.2		12.5	
**Age (years)**			**<0.001**		**<0.001**		**<0.001**
20-44	15,859 (42.6)	8.0		1.7		2.3	
45-64	12,316 (33.0)	33.1		7.1		11.7	
≥65	9,100 (24.4)	52.5		8.8		22.0	
**Gender**			**<0.001**		**<0.001**		**<0.001**
Men	17,419 (46.7)	23.3		4.6		7.7	
Women	19,856 (53.3)	30.5		5.7		12.4	
**Race/ethnicity**			**<0.001**		**<0.001**		**<0.001**
Mexican-American	6,047 (16.2)	18.9		4.6		4.9	
Other Hispanic	2,953 (7.9)	23.4		5.4		7.2	
Non-Hispanic White	17,246 (46.3)	32.8		4.8		14.6	
Non-Hispanic Black	7,633 (20.5)	27.4		7.6		7.2	
Other races	3,396 (9.1)	15.9		2.9		6.7	
**PIR**			**<0.001**		**<0.001**		**<0.001**
0-129%	11,139 (29.9)	28.7		7.0		8.6	
≥130%	26,136 (70.1)	26.5		4.5		10.9	
**Health insurance status**			**<0.001**		**<0.001**		**<0.001**
No	7,384 (19.8)	13.0		3.1		3.5	
Yes	29,891 (80.2)	30.7		5.7		11.9	
**Smoking status**			**<0.001**		**<0.001**		**<0.001**
Never	20,346 (68.4)	22.8		4.2		8.8	

Former	9,401 (31.6)	37.5		6.6		15.1	
Current	7,528 (25.3)	26.2		6.2		7.9	
**Diabetes**			**<0.001**		**<0.001**		**<0.001**
No	31,904 (85.6)	23.8		4.4		9.1	
Yes	5,357 (14.4)	47.2		10.0		16.9	
Don't know	14 (0.0)	21.4		7.1		7.1	
**BMI (kg/m^2)**			**<0.001**		**<0.001**		**<0.001**
<25	10,825 (29.0)	19.7		3.9		7.3	
25≤BMI<30	12,552 (33.7)	25.8		4.7		9.8	
≥30	13,898 (37.3)	34.2		6.7		12.9	
**Central obesity**			**<0.001**		**<0.001**		**<0.001**
Yes	15,403 (42.4)	17.4		6.4		12.9	
No	20,900 (57.6)	33.7		3.5		6.1	
**Type of arthritis**			*NA*		*NA*		*NA*
RA	1,943 (5.2)	*NA*		*NA*		*NA*	
OA	3,804 (10.2)	*NA*		*NA*		*NA*	
Others	1,200 (3.2)	*NA*		*NA*		*NA*	
Refused or don't know	3,184 (8.5)	*NA*		*NA*		*NA*	

Note: Boldface indicates statistical significance (*P*<0.05).

[a] Values in columns "No" and "Yes" expressed as %. OA, osteoarthritis; PIR, poverty-to-income ratio; RA, rheumatoid arthritis.

[b] *P* values estimated using Chi-square tests.

[c] Fisher's exact test was performed for diabetes.

Appendix Table 2. Associations between ultra-processed food (UPF) intake and arthritis in sensitivity analyses.[a,b]

Models[c]	Q1	Q2	Q3	Q4	*P*-trend	Continuous, *P* value
**Overall arthritis**						
Model S1	Ref	0.00 (0.00, 0.00)	0.00 (0.00, 0.00)	2.44 (1.13, 5.27)	0.11	0.65 (0.56, 0.76), **<0.001**
Model S2	Ref	0.00 (0.00, 0.00)	0.00 (0.00, 0.01)	1.89 (0.89, 4.01)	0.11	1.38 (1.17, 1.62), **<0.001**
Model S3 stratified by gender[d]						
Men	Ref	1.16 (0.99, 1.35)	1.21 (1.02, 1.42)	1.22 (1.02, 1.45)	**0.03**	1.03 (1.00, 1.06), **0.04**
Women	Ref	1.09 (0.95, 1.25)	1.18 (1.03, 1.36)	1.25 (1.10, 1.41)	**<0.001**	1.04 (1.02, 1.06), **<0.001**
Model S4	Ref	1.08 (0.99, 1.19)	1.11 (1.00, 1.24)	1.13 (1.01, 1.26)	**0.03**	1.02 (1.00, 1.04), **0.04**
Model S5	Ref	1.10 (0.99, 1.21)	1.15 (1.03, 1.28)	1.16 (1.04, 1.29)	**0.01**	1.03 (1.01, 1.04), **0.01**
Model S6	Ref	1.11 (1.01, 1.22)	1.18 (1.06, 1.31)	1.21 (1.09, 1.35)	**<0.001**	1.03 (1.02, 1.05), **<0.001**
Model S7 stratified by diabetes[e]						
Yes	Ref	1.21 (0.98, 1.49)	1.49 (1.18, 1.87)	1.14 (0.89, 1.45)	0.15	1.03 (0.98, 1.07), 0.23
No	Ref	1.09 (0.98, 1.22)	1.13 (1.01, 1.26)	1.23 (1.10, 1.37)	**<0.001**	1.04 (1.02, 1.06), **<0.001**
**RA**						
Model S1	Ref	0.00 (0.00, 0.03)	0.00 (0.00, 0.02)	2.82 (0.70, 11.41)	0.21	1.12 (0.83, 1.51), 0.46
Model S2	Ref	0.00 (0.00, 0.04)	0.00 (0.00, 0.03)	2.86 (0.71, 11.52)	0.24	1.61 (1.16, 2.23), **0.005**
Model S3 stratified by gender						
Men	Ref	1.21 (0.81, 1.79)	1.17 (0.83, 1.66)	1.24 (0.88, 1.76)	0.27	1.04 (0.98, 1.10), 0.19
Women	Ref	1.18 (0.89, 1.57)	1.38 (1.06, 1.79)	1.49 (1.16, 1.90)	**<0.001**	1.07 (1.02, 1.11), **0.003**
Model S4	Ref	1.19 (0.95, 1.47)	1.25 (1.02, 1.53)	1.33 (1.09, 1.64)	**0.01**	1.05 (1.01, 1.08), **0.01**
Model S5	Ref	1.21 (0.97, 1.50)	1.26 (1.03, 1.55)	1.36 (1.10, 1.67)	**0.01**	1.05 (1.02, 1.09), **0.01**
Model S6	Ref	1.20 (0.96, 1.49)	1.28 (1.05, 1.57)	1.37 (1.12, 1.68)	**0.002**	1.05 (1.02, 1.09), **0.003**
Model S7 stratified by diabetes						
Yes	Ref	1.27 (0.87, 1.85)	1.63 (1.14, 2.35)	1.29 (0.92, 1.81)	0.06	1.04 (0.99, 1.09), 0.11
No	Ref	1.18 (0.92, 1.52)	1.21 (0.96, 1.53)	1.41 (1.11, 1.79)	**0.01**	1.06 (1.02, 1.10), **0.01**
**OA**						
Model S1	Ref	0.00 (0.00, 0.01)	0.00 (0.00, 0.01)	3.95 (1.20, 12.96)	0.06	0.44 (0.35, 0.55), **<0.001**
Model S2	Ref	0.00 (0.00, 0.01)	0.00 (0.00, 0.01)	2.38 (0.73, 7.69)	0.07	0.95 (0.73, 1.25), 0.73
Model S3 stratified by gender						
Men	Ref	1.03 (0.78, 1.35)	1.07 (0.83, 1.38)	1.00 (0.73, 1.38)	0.91	1.00 (0.95, 1.06), 0.93
Women	Ref	0.98 (0.81, 1.19)	1.03 (0.86, 1.23)	1.14 (0.95, 1.37)	0.13	1.03 (1.00, 1.06), 0.08
Model S4	Ref	0.96 (0.84, 1.10)	0.98 (0.84, 1.14)	1.01 (0.85, 1.20)	0.87	1.00 (0.97, 1.03), 0.80

Model S5	Ref	0.98 (0.85, 1.13)	1.02 (0.88, 1.20)	1.03 (0.86, 1.22)	0.68	1.01 (0.98, 1.04), 0.60
Model S6	Ref	0.99 (0.86, 1.14)	1.03 (0.89, 1.20)	1.08 (0.91, 1.27)	0.33	1.02 (0.99, 1.05), 0.26
Model S7 stratified by diabetes						
Yes	Ref	0.98 (0.73, 1.30)	1.12 (0.85, 1.46)	1.00 (0.70, 1.42)	0.81	1.01 (0.95, 1.07), 0.74
No	Ref	0.99 (0.85, 1.16)	1.01 (0.86, 1.19)	1.09 (0.92, 1.30)	0.32	1.02 (0.99, 1.05), 0.28

Note: Boldface indicates statistical significance (*P*<0.05).

[a] Cells indicate survey-weighted odds ratios (95% confidence intervals) and *P* values. OA, osteoarthritis; RA, rheumatoid arthritis; TEI, total energy intake.

[b] Q2-Q4 indicate quartiles of ultra-processed food (UPF) intake expressed as a percentage of total energy intake (%TEI) or a percentage of total food grams intake (%g/d) (Q1: reference group).

For %TEI, Q1: %TEI≤40.79, Q2: 40.79<%TEI≤55.17, Q3: 55.17<%TEI≤69.41, Q4: %TEI>69.41.

For %g/d, Q1: %g/d≤14.04, Q2: 14.04<%g/d ≤26.38, Q3: 26.38<%g/d ≤45.48, Q4: %g/d >45.48.

[c] Model S1: Outcome ~ UPF (%g/d)

Model S2: Outcome ~ UPF (%g/d) + age + gender + race/ethnicity + poverty-to-income ratio + health insurance status + smoking status

Model S3: Outcome ~ UPF (%TEI) + age + race/ethnicity + poverty-to-income ratio + health insurance status + smoking status

Model S4: Outcome ~ UPF (%TEI) + age + gender + race/ethnicity + poverty-to-income ratio + health insurance status + smoking status + continuous BMI

Model S5: Outcome ~ UPF (%TEI) + age + gender + race/ethnicity + poverty-to-income ratio + health insurance status + smoking status + central obesity

Model S6: Outcome ~ UPF (%TEI) + age + gender + race/ethnicity + poverty-to-income ratio + health insurance status + smoking status + diabetes

Model S7: Outcome ~ UPF (%TEI) + age + gender + race/ethnicity + poverty-to-income ratio + health insurance status + smoking status

[d] *P*=0.63, 0.44, and 0.77 for the interaction between gender and UPF intake for arthritis, RA and OA, respectively.

[e] *P*=0.29, 0.60, and 0.47 for the interaction between diabetes and UPF intake for arthritis, RA and OA, respectively.
